# Ethnic Aspects of Valproic Acid P-Oxidation

**DOI:** 10.3390/biomedicines12051036

**Published:** 2024-05-08

**Authors:** Natalia A. Shnayder, Violetta V. Grechkina, Vera V. Trefilova, Mikhail Ya. Kissin, Ekaterina A. Narodova, Marina M. Petrova, Mustafa Al-Zamil, Natalia P. Garganeeva, Regina F. Nasyrova

**Affiliations:** 1 Institute of Personalized Psychiatry and Neurology, Shared Core Facilities, V.M. Bekhterev National Medical Research Centre for Psychiatry and Neurology, 192019 Saint Petersburg, Russia; grechkina.vv@mail.ru (V.V.G.); vera.v.trefilova@yandex.ru (V.V.T.); 2 Shared Core Facilities “Molecular and Cell Technologies”, V.F. Voino-Yasenetsky Krasnoyarsk State Medical University, 660022 Krasnoyarsk, Russia; katya_n2001@mail.ru (E.A.N.); stk99@yandex.ru (M.M.P.); 3 Department of Psychiatry and Addiction, I.P. Pavlov First St. Petersburg State Medical University, 197022 Saint Petersburg, Russia; kissin.m@yandex.ru; 4 Department of Physiotherapy, Faculty of Continuing Medical Education, Peoples’ Friendship University of Russia, 117198 Moscow, Russia; alzamil@mail.ru; 5 Department of General Medical Practice and Outpatient Therapy, Siberian State Medical University, 634050 Tomsk, Russia; garganeeva@gmail.com; 6 International Centre for Education and Research in Neuropsychiatry, Samara State Medical University, 443016 Samara, Russia

**Keywords:** valproic acid, metabolism, pharmacokinetics, pharmacogenetics, P-oxidation, ethnos, adverse drug reaction, risk factor, personalized approach, metabolite, biomarker, single-nucleotide variant

## Abstract

The safety of the use of psychotropic drugs, widely used in neurological and psychiatric practice, is an urgent problem in personalized medicine. This narrative review demonstrated the variability in allelic frequencies of low-functioning and non-functional single nucleotide variants in genes encoding key isoenzymes of valproic acid P-oxidation in the liver across different ethnic/racial groups. The sensitivity and specificity of pharmacogenetic testing panels for predicting the rate of metabolism of valproic acid by P-oxidation can be increased by prioritizing the inclusion of the most common risk allele characteristic of a particular population (country).

## 1. Introduction

**Figure 1:**
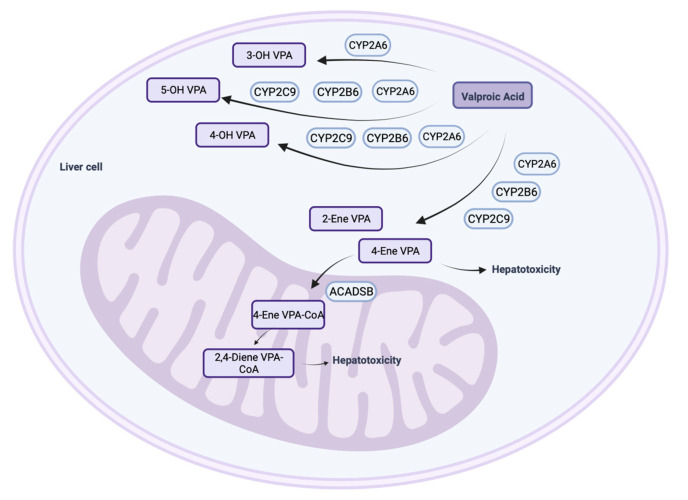
P-oxidation of valproic acid in human hepatocytes (created with BioRender.com: https://www.biorender.com/ (accessed on 12 October 2023)). Note: 3-OH VPA—3-hydroxy valproic acid; 4-OH VPA—4-hydroxy valproic acid; 5-OH VPA—5-hydroxy valproic acid; 2-ene VPA—2-ene valproic acid; 4-ene VPA—4-ene valproic acid; ACADB—acyl coenzyme dehydrogenase short/branched chain; 4-ene VPA-CoA—4-ene valproic acid coenzyme A; 2,4-ene VPA-CoA—2,4-ene valproic acid coenzyme A.

Valproic acid (CH3CH2CH2)2CHCOOH 2-propylpentanoic acid, VPA) is one of the most prescribed psychotropic drugs in neurology and psychiatry [1,2]. Its effectiveness and safety varied between patients, which is explained by individual pharmacokinetics (in particular, the rate of metabolism of VPA and its active metabolites in the liver). VPA metabolism and genetic biomarkers associated with changes in VPA pharmacokinetics continue to be actively studied [3]. Three main pathways of VPA biotransformation are known: P-oxidation via cytochrome 450 isoenzymes (CYPs); glucuronidation via uridylglucuronyltransferase (UGT) isoenzymes; and acetylation in the tricarboxylic acid cycle (mitochondrial oxidation) (Figure 1) [1,4,5].

CYPs play a critical role in the P-oxidation of VPA in hepatocytes [1]. At the same time, their active metabolites (4-hydroxy(OH)-VPA and 5-OH-VPA) are formed with the participation of isoenzymes CYP2C9, CYP2B6, and CYP2A6, although other CYPs (CYP2C19, CYP2D6, etc.) also take part in the P-oxidation of VPA. The isoenzymes CYP2A6 and CYP2B6 together provide the formation of 20–25% of 4-ene-VPA, 4-OH-VPA, and 5-OH-VPA. The CYP2A6 isoenzyme mediates the oxidation of VPA to 3-OH-VPA, but with polytherapy (for example, when VPA is co-administered with strong CYP2A6 inhibitors), the formation of 3-OH-VPA in human liver microsomes is inhibited [1].

The purpose of this narrative review is to update knowledge about the ethnic aspects of VPA P-oxidation from the perspective of personalized medicine.

## 2. Effect of Variable Alleles of Genes Encoding Cytochrome P450 Isoenzymes on the P-Oxidation Rate of Valproic Acid

**Figure 2:**
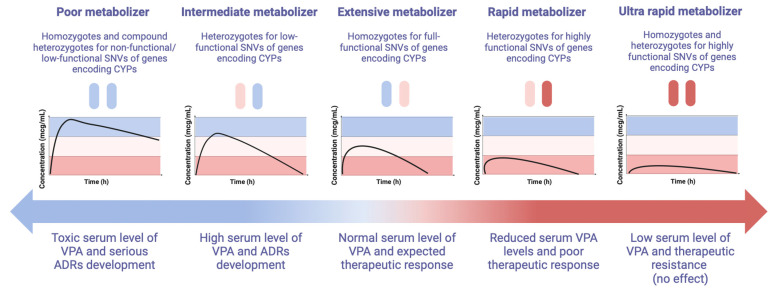
Possible phenotypes of patients receiving valproic acid depending on the results of pharmacogenetic profiling (created with BioRender.com: https://www.biorender.com/ (accessed on 16 January 2024)). The normal range for VPA is 50–100 mcg/mL; the high range is 100–120 mcg/mL; the toxic range is >120 mcg/mL; the reduced range is 30–50 mcg/mL; and the low level is <30 mcg/mL. Note: ADRs—adverse drug reactions, CYP—cytochrome P450; VPA—valproic acid; SNVs—single nucleotide polymorphisms.

Preclinical studies of the pharmacokinetics and pharmacogenetics of VPA in vitro and in vivo, as well as clinical studies [6], have confirmed that the rate of P-oxidation of VPA is associated with the expression and functional activity of CYPs, but interindividual variability in these characteristics is genetically determined. In particular, different allelic variants (fully functional, non-functional, low functional, or high functional) of genes encoding CYPs can significantly influence interindividual variability in the rate of VPA P-oxidation in the liver, which explains the importance of a personalized approach to the selection and dosing of valproate. Depending on the results of patient pharmacogenetic profiling (PGx), usually, five phenotypes have been proposed: a general or extensive metabolizer (EM); an intermediate metabolizer (IM); a poor metabolizer (PM); a rapid metabolizer (RM); and an ultra-rapid metabolizer (URM) (Figure 2). However, the types of metabolizers vary depending on the consortium; not all share the types of EM and RM. For example, the Clinical Pharmacogenetics Implementation Consortium (CPIC) [7], which is a joint project of PharmGKB and the Pharmacogenomics Global Research Network (PGRN), identifies five grades, and the Dutch Pharmacogenetics Working Group (DPWG) identifies only four types of metabolizers: a normal metabolizer (NM), IM, PM, and URM [8].

The PM group is persons who lack a fully functional enzyme or have significantly reduced (usually more than 50% compared to EM) CYP isoenzyme expression and, consequently, there is a significant decrease in the metabolic rate of VPA and an increase in its level in the blood and duration of exposure in other tissues in the human body. Such a person will need lower doses of VPA (usually 50% lower than the average therapeutic dose or the cancellation of the admission of this drug) [9], which is metabolized by this enzyme. In the case of prescribing standard doses of VPA, such patients are more likely to experience adverse drug reactions (ADRs) and toxicity (hepatotoxicity, neurotoxicity, etc.) [1,3].

The IM group is very heterogeneous since the decrease in the activity of the CYP isoenzyme can vary from 10 to 50%, but on average by 25% due to the carriage of one non-functional or low-functional allele of one or more key genes responsible for the P-oxidation of VPA [3]. In this regard, the level of VPA in the blood serum will vary in different patients, depending on the effect of a particular SNV on a decrease in the functional activity and/or the expression of a particular CYP isoenzyme or several isoenzymes involved in the P-oxidation of VPA. It should be recognized that this issue is debatable and needs further study. Nevertheless, a moderate reduction in the dose of VPA is required when prescribed in monotherapy to patients with the IM phenotype (usually up to 25% of the recommended average therapeutic dose) under the control of TDM [9]. Over 600 drugs have reported interactions with VPA. However, when co-prescribing VPA with inhibitors of a key low-functioning isoenzyme, it may be necessary to reduce the dose by 50% or abandon the co-administration of drugs.

Patients using multiple medications may require more frequent evaluation of VPA levels due to potential drug–drug interactions. Furthermore, most VPA is bound to protein in the blood. However, it is the unbound portion of the drug that is the active component [10]. Therefore, a patient with a condition resulting in lower-than-normal amounts of protein in their blood may be at increased risk of having a supratherapeutic or excess amount of circulating VPA and, therefore, require TDM of both total and free VPA levels [11]. For example, TDM results may be false positive in vegetarians, patients with eating disorders, pregnant women with gestosis of the first trimester of pregnancy, or representatives of certain ethnic groups with a habitually low intake of animal proteins. In such cases, the level of the free fraction of VPA in plasma is explained by the fact that the clearance of VPA is low (6–20 mL/h/kg) due to its high binding to proteins (87–95%) [4,12].

Minimum VPA levels are more accurate when measured immediately before the next dose is administered. After taking a double dose per day, the time of taking a blood sample is important and affects the correct interpretation of the therapeutic and toxic levels of VPA. However, there is no consensus yet on when we would depend on the optimal time to assess the minimum levels of delayed-release VPA drugs (prolonged VPA), which affects the correct clinical interpretation of the serum concentration of VPA. As a rule, when taking prolonged VPA once a day in the morning, it is expected that in a blood sample taken 21–24 h after taking the first dose of the drug, the concentration is within 3% of the minimum value. Conversely, a blood test 12–15 h after the last dose will give a value that is 18–25% higher than the actual minimum value [13]. Measuring serum VPA levels at 24 h is recommended and provides a more accurate value for patients on a maximum daily dose or overdose. Measuring it at 12 h in case of a maximum daily dose or overdose would give a 1.3 times higher value than the actual trough value. Trough sampling is easily achieved just before a morning daily dose, but the issue arises when the patient receives a night dose because collecting a blood sample 21–24 h later may be limited by the operational hours of the laboratory. To avoid patients’ inconvenience, serum VPA levels can be tested at 12 h, though they have to be reduced by 1.3 times to calculate the accurate trough levels [11].

Such complex relationships of the genotype and phenotype of CYP isoenzymes of the P-oxidation of VPA among different people, even within the same population and even more so in different populations, make a personalized medical approach to the selection of valproate pharmacotherapy a difficult task.

The VPA level measures the amount of VPA in the blood: either the total level or the free level of the drug. Monitoring of serial VPA levels is required to maintain the drug within the narrow recommended therapeutic range. Subtherapeutic levels place the patient at risk of recurrence of the condition for which they are taking VPA, and supratherapeutic (or high) and toxic levels place the patient at risk of ADRs and toxic side effects. It is generally considered that the therapeutic range for VPA is 50–100 mcg/mL, and the toxic level is >120 mcg/mL [14]. Within the therapeutic (normal) range, most patients expected a therapeutic response to VPA without serious ADRs. However, even within the recommended therapeutic range, the results differ in individual patients, and some may experience VPA-induced ADRs at serum levels in the lower part of the spectrum (according to TDM data) [15,16]. The range of efficacy and ADRs observed with the use of this drug requires not only TDM serial levels of VPA [5,9] to ensure optimal dosage for each individual patient but also pharmacometabolic testing of the level of toxic metabolites of VPA [1].

The high level of VPA in the blood according to TDM data and, accordingly, the risk of developing ADRs will largely depend on the homozygous or compound heterozygous carrier of non-functional alleles of the *CYP2C9*, *CYP2A6,* and *CYP2B6* genes. This is due to the fact that CYPs encoded by these genes are considered to be one of the main “players” in the P-oxidation of VPA, especially in patients with impaired glucuronidation of this drug [1]. However, the most studied SNVs are the *CYP2C9* and *CYP2A6* genes. For example, the major (or wild-type) allelic variant CYP2C9*1 of the *CYP2C9* gene, encoding the fully functional CYP2C9 isoenzyme, is associated with an active process of formation of 4-OH-VPA and 5-OH-VPA by 75–80% [17]. On the contrary, in homozygotes or compound heterozygotes for non-functional allelic variants of the *CYP2C9* gene (for example, CYP2C9*2, CYP2C9*3, CYP2C9*6, etc.), the biotransformation of VPA is significantly slowed down, which is associated with an increase in the level of VPA in the blood to toxins and the development of VPA-induced ADRs [18]. Despite the fact that VPA has linear pharmacokinetics and a wide reference range in the blood (according to TDM data), most associative genetic studies indicate that in patients with the PM phenotype, the level of VPA can increase significantly (two times higher than in patients with the EM phenotype) [19,20,21]. Therefore, the predictive PGx of VPA [22] used to identify patients with PM and IM phenotypes before or at the start of prescribing this drug may help with choosing the timing and frequency of TDM since the therapeutic serum level (and even supratherapeutic or toxic levels) can be achieved earlier than the expected time to reach the therapeutic level with dose titration in patients with the EM phenotype [9].

Also, allelic variants of the *CYP2A6* gene play an important role in changing the pharmacokinetics of VPA because the CYP2A6 isoenzyme encoded by it contributes about 50% to the formation of 3-OH-VPA [1]. In particular, homozygous carriers of the highly functional CYP2B6*4 allele (rs2279343; 785A>G) have a URM phenotype and a higher rate of VPA oxidation, which are associated with therapeutic resistance to valproate. In contrast, patients with the PM phenotype who are homozygous or compound heterozygous carriers of non-functional *CYP2B6* gene SNVs (e.g., rs3745274, 516G>T, or rs2279343, 785A>G) had higher plasma VPA levels [1], which was associated with a high risk of the accumulation of VPA in the human body, the development of chronic intoxication, and ADRs [5].

However, genetically determined interindividual variability in VPA pharmacokinetics associated with allelic variants in the CYP genes may vary among different ethnic and racial groups of patients with neurological diseases and mental disorders [1,23,24,25]. This largely depends on the allelic frequency of non-functional and low-functional single nucleotide variants (SNVs) of CYP family genes in different populations, which is important to consider when developing PGx panels and their use in real-life clinical practice around the world [2,26]. The use in PGx panels of studies of allelic variants of *CYP* genes that have demonstrated a role in altering VPA pharmacokinetics and a significance in the prediction and prevention of VPA-induced ADRs in one ethnic group may not be clinically or economically feasible in another ethnic group. It is likely that PGx panels that assess the contribution of CYPs to safety assessments cannot be universal but need to be tailored to specific populations.

## 3. Cytochrome P450-Catalyzed Oxidation

Humans are exposed to a wide range of xenobiotics throughout life (from newborn to old age), including environmental substances, drugs, and nutrients, which can cause disturbances in cellular metabolism and negatively affect human health. The central link in protection against the negative effects of xenobiotics is a unique hemoprotein, cytochrome P450 [27,28,29], which explains the interest of scientists and clinicians in studying the role of CYP isoenzymes in the pharmacokinetics of VPA.

It is known that CYP is a heme-containing protein characterized by a maximum absorption wavelength of 450 nm in the reduced state in the presence of carbon monoxide [27]. Isoenzymes of the CYP superfamily (CYPs) contain 400–500 amino acid residues and one heme prosthetic group in the active site [27]. Iron in its ferrous form (Fe^3+^) can exist in two spin states [27]. When a substrate (e.g., VPA) binds to a CYP, an iron–water molecule is displaced from the CYP, changing Fe^3+^ from a sixfold to a fivefold coordination state in which Fe^3+^ moves out of the plane of the heme ring [27]. Most reactions of CYPs are oxidations and involve the use of molecular oxygen (O_2_), acting as monooxygenases or mixed oxidases with pyridine nucleotide nicotinamide adenine dinucleotide (NADH), or nicotinamide adenine dinucleotide phosphate (NADPH) can be a cofactor [30].

CYPs are divided into two main classes: (1) CYPs that are involved in the detoxification of xenobiotics and (2) CYPs that are involved in the biosynthesis of endogenous compounds. It is known that CYPs play a central role in cellular metabolism and the maintenance of cellular homeostasis; they are expressed in all tissues in the human body (mainly in the liver and small intestine) [27]. To a lesser extent, they are expressed in the inner mitochondrial membranes of steroidogenic tissues (adrenal cortex, testes, ovaries, mammary gland, and placenta) [27,31]. CYPs play an important role in the P-oxidation of unsaturated fatty acids and cholesterol biosynthesis and the metabolism of xenobiotics in the microsomal fraction of the liver [27]. They participate in the synthesis and degradation of endogenous steroid hormones [27] and the metabolism of vitamins [27]. In the brain, CYPs are involved in the regulation of endogenous gamma-aminobutyric acid receptor (GABA) receptor agonists, the maintenance of cholesterol homeostasis, and the elimination of retinoids [27]. The CYP 1–4 families (e.g., 1A2, 2C9, 2C19, 2D6, 2E1, and 3A4) catalyze P-oxidation reactions associated with the detoxification of drugs and other xenobiotics in the liver, and other families have important endogenous functions [27].

Genes encoding isoenzymes of the CYP superfamily include more than 13,000 genes representing more than 400 gene families. In humans, at least 57 different *CYP* genes and 58 pseudogenes, 18 different families, and 44 subfamilies have been identified [27]. The most studied genes encoding CYPs are involved or likely to be involved in the metabolism of VPA and its compounds.

## 4. Risk Factors for the Impaired P-Oxidation of Valproic Acid

The expression and function of CYPs involved in the P-oxidation of VPA can be influenced by various factors, including physiological factors (age, sex, hormones, environment, allelic variants of *CYP* family genes, drug use, drug–drug interactions) and pathological conditions (e.g., inflammation and cholestasis) [27]. These factors determine the clinical manifestations of VPA-induced ADRs, which arise as a consequence of a violation of its P-oxidation.

**Figure 3:**
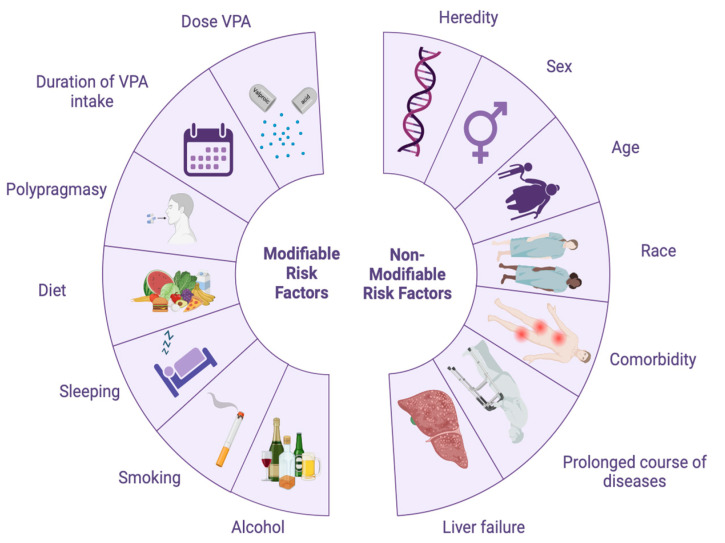
Diagram of the main risk factors for the impaired P-oxidation of valproic acid (VPA) in human hepatocytes (created with BioRender.com: https://www.biorender.com/ (accessed on 16 January 2024)).

Risk factors that can influence VPA P-oxidation can be divided into modifiable (they can be controlled or changed) and non-modifiable (they cannot be controlled) (Figure 3, Table 1). If a patient has two or more risk factors, then the likelihood of developing VPA-induced ADRs is higher. For example, modifiable risk factors for VPA-induced ADRs include smoking [32,33], obesity [34], low physical activity [35], and poor diet [36]. Non-modifiable risk factors for VPA-induced ADRs include heredity [37,38], gender [39,40], race and ethnicity [41], age [42,43], liver failure [44], liver disease [44,45], and other comorbid diseases [46].

In conclusion, the ethnic and racial origin of patients with neurological diseases and mental disorders are non-modifiable risk factors for impaired VPA P-oxidation. This risk, in turn, depends on the allelic frequency of non-functional/low-functioning SNVs of *CYP* family genes in different populations.

## 5. Valproic Acid P-Oxidation and Ethnicity

The results of association genetic studies and genome-wide studies indicate that it is not race and ethnicity but the allelic frequency of low-functional and non-functional SNVs of genes encoding key isoenzymes of the CYP family that are associated with a decrease in the rate of P-oxidation of VPA in various organs and tissues, primarily in the liver [47,48]. In addition, the implementation of SNVs into a pathological phenotype (as a multifactorial disorder) occurs with additional exposure to environmental factors: physical, chemical, food, and environmental (environmental pollution) [31]. Under certain environmental conditions, certain SNVs may either predispose or prevent VPA-induced toxicity [31,49] and the development of ADRs.

As is known, SNVs are differences in the DNA sequence of one nucleotide that occur in a significant part (more than 1%) of a large population as a result of point mutations. They can be identified within gene coding sequences (exons), in non-coding regions (introns, promoters), or in intergenic regions [50,51]. SNVs can influence gene transcription and translation quantitatively or qualitatively and determine protein structure and function (e.g., CYPs) [52]. SNVs that are localized in exons predominantly affect the functional activity of CYPs, leading to their decrease (risk of developing VPA-induced ADRs) or increase (risk of therapeutic resistance). SNVs that are located in introns or promoters are associated with a decreased expression of CYPs (risk of developing VPA-induced ADRs) or an increased expression of CYPs (risk of VPA therapeutic resistance) [53,54]. Although most SNVs do not directly or significantly contribute to the pathological phenotypes of IM and PM in patients receiving VPA [55,56], the risk of developing VPA-induced ADRs (e.g., hepatotoxicity [1,19], neurotoxicity [57], teratogenicity [58], metabolic syndrome [2]) may be variable (low, moderate, or high) in representatives of different populations, depending on genetically determined changes in enzymatic activity and/or the expression of CYPs involved in the P-oxidation of VPA. This is important to consider in the development of PGx panels for various ethnic and racial groups of patients and their use in the real clinical practice of a neurologist or psychiatrist [59,60].

The molecular mechanisms responsible for ethnic differences in VPA metabolism are explained by advances in molecular biology and population genetic studies in recent years [61,62]. In particular, significant differences in the population frequency of some variative (“mutant” or minor) SNVs in different countries of the world have been shown, which cause differences in the prevalence of patients with neurological diseases and mental disorders who are homozygous and heterozygous for the minor allele(s) of the gene(s) of the CYP family and associated with the impaired P-oxidation of VPA in the liver and the development of ADRs [63,64,65,66,67,68] (Table 2).

### 5.1. The CYP2A6 Gene

**Figure 4:**
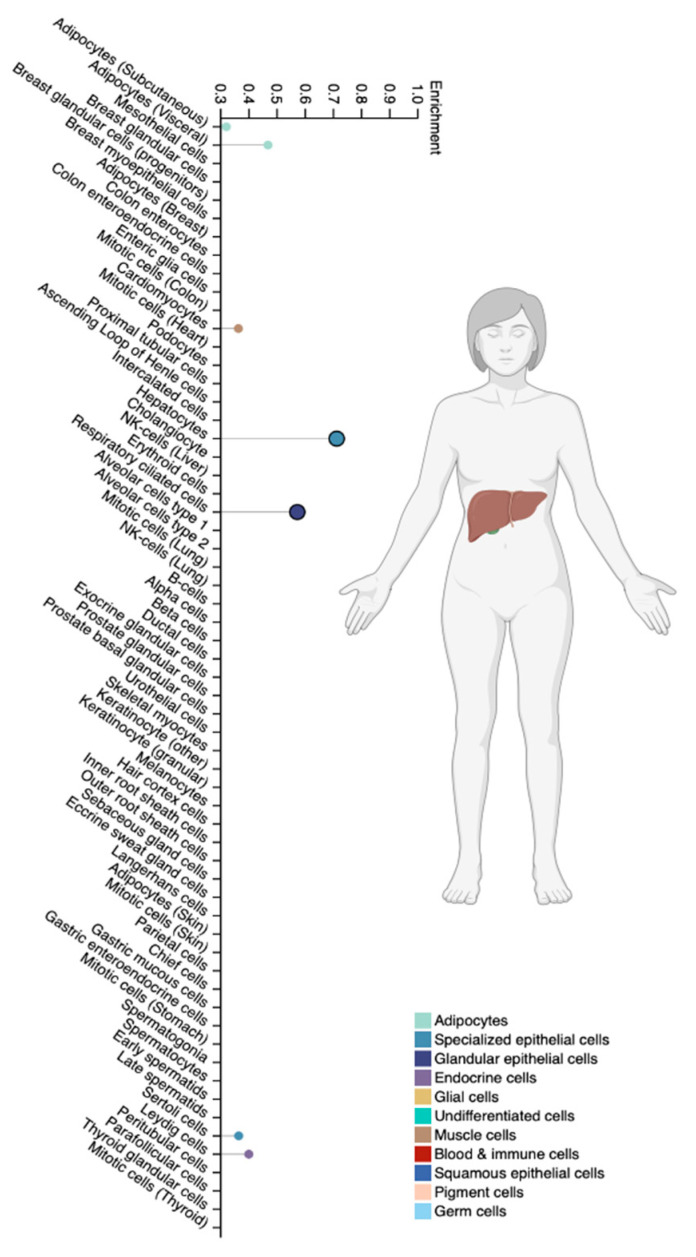
Tissue-specific cell types for the CYP2A6 isoenzyme (modified by the authors of The Human Protein Atlas [69]).

The CYP2A6 isoenzyme is a member of the cytochrome P450 superfamily family 2 subfamily A member 6 and is expressed predominantly in hepatocytes and specialized extrahepatic cell types [25] (Figure 4).

The *CYP2A6* gene is located on chromosome 19 at position 19q13.2 (OMIM 122720) and encodes the CYP2A6 protein, consisting of 494 amino acids [70]. CYP2A6 accounts for about 4% of the total CYP450 content, and significant differences in CYP2A6 activity are mainly associated with SNVs [70]. VPA and other drugs (e.g., halothane, methoxyflurane, disulfiram, coumarin, and losigamon) are metabolized by CYP2A6 [71]. The CYP2A6 isoenzyme is the most active in the metabolism of VPA by 3-hydroxylation to form 3-OH-VPA [72].

The most studied SNVs of the *CYP2A6* gene are CYP2A6*2 (rs1801272, 479T>A), CYP2A6*4 (gene deletion), CYP2A6*5 (rs5031017, 1436G>T), and CYP2A6*20 (rs28399444). These SNVs are associated with a decrease or shutdown of CYP2A6 enzyme activity and have different allelic frequencies among representatives of different ethnic groups [71]. Patients with heterozygous (CYP2A6*1/*4) or homozygous (CYP2A6*4/*4) genotypes, known as PM, had higher plasma VPA concentrations than URM and, therefore, had a higher risk of VPA accumulation and development of VPA-induced ADRs [1,73].

Less studied low-function SNVs that are associated with reduced activity of this isoenzyme include *7 (rs5031016, g.6558 T>C), *10 (rs28399468, g.6600G>T) *11 (rs111033610. g.3391T>C), *17 (rs28399454, g.5065G>A), *18 (rs1809810 g.5668A>T), and и *19 (rs5031016 g.6558T>C) [70]. The prevalence of CYP2A6 PMs in the Caucasian population is ≤1% [71]. The deletion of the *CYP2A6* gene is very common in Asian patients (up to 20%), which explains the particular importance of therapeutic drug monitoring (TDM) and PGx in patients receiving VPA [70,71] in this ethnic group. The low-functioning allele CYP2A6*9 (rs28399433) is common in all major populations (allele frequency ranging from 8% in Africans to 23% in East Asians), while allelic variants CYP2A6*17 (rs28399454), CYP2A6*23 (rs56256500), CYP2A6*25, and CYP2A6*28 are found only in Africans, and CYP2A6*7 (rs5031016) and CYP2A6*19 (rs1809810) are found predominantly in East Asian populations. In Europeans, the most common low-functioning allelic variants are CYP2A6*9 (s28399433) and CYP2A6*35 (rs143731390) (allelic frequency range 11% and 15%, respectively) [73].

Since the CYP2A6 isoenzyme is a high-affinity isoenzyme of both nicotine and its oxidized metabolite (cotinine), allelic variants of the *CYP2A6* gene are also being studied for the treatment of tobacco abuse [71] and, therefore, may be important in smokers receiving VPA, which is of undoubted clinical interest.

### 5.2. The CYP2B6 Gene

**Figure 5:**
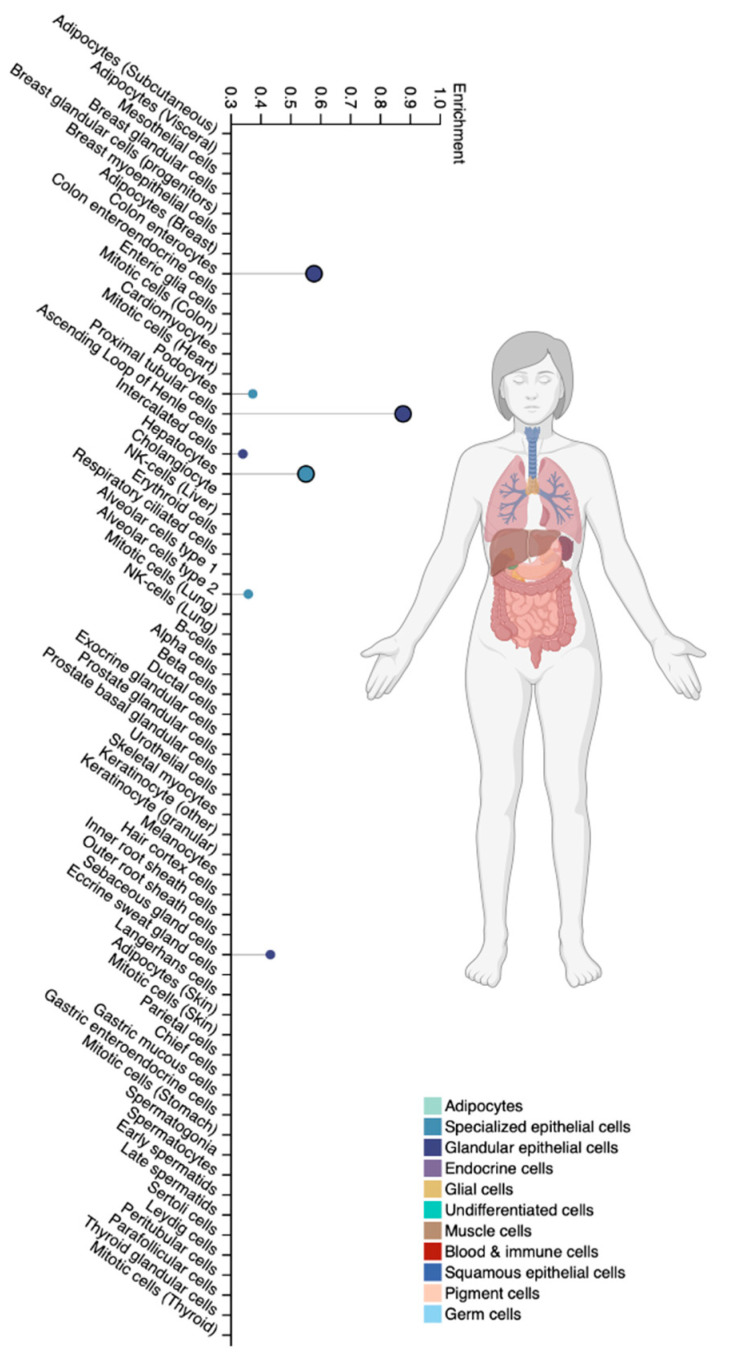
Tissue-specific cell types for the CYP2B6 isoenzyme (modified by the authors of The Human Protein Atlas [69]).

The CYP2B6 isoenzyme is cytochrome P450 family 2 subfamily B member 6 [74]. This isoenzyme is highly expressed in the liver and to a certain extent in extrahepatic tissues, such as the brain, kidneys, digestive tract, and lungs [75,76] (Figure 5). The expression of CYP2B6 varies greatly among different ethnic groups due to non-genetic factors, SNVs, inducibility, and irreversible inhibition by various endogenous and exogenous compounds [77]. CYP2B6 accounts for up to 10% of functional CYP isoenzymes in the liver [76]. At the same time, the enzymatic activity of CYP2B6 is 1.7 times higher in women compared to men [78]. CYP2B6 is known to be an active catalyst for the formation of 4-ene-VPA, 4-OH-VPA, and 5-OH-VPA [72], which have both therapeutic and (mainly) toxic effects. In addition to VPA, CYP2B6 is involved in the metabolism of bupropion, cyclophosphamide, efavirenz, ifosfamide, ketamine, and methadone [75,77].

The human *CYP2B6* gene is located on chromosome 19 at position 19q13.2 (OMIM 123930) and is highly polymorphic [79]. The CYP2B6*2 allelic variant is more common in Europe and Africa compared to Asian populations [77], and the CYP2B6*4 variant is more common in Africans, Americans, and Asians compared to European populations. Also, there is intraethnic and interethnic variability in the frequency of low-functional allelic variants of CYP2B6*6 and CYP2B6*18. Thus, the allelic frequency of CYP2B6*6 ranges from 0.33 to 0.5 in African Americans and Africans, 0.10–0.21 in Asians, 0.14–0.27 in Caucasians, and 0.62 in residents of Papua New Guinea [77], although the allelic frequency may have intraethnic differences within a particular racial/ethnic group [77]. For example, the frequency of the CYP2B6*6 variant in the Nigerian Yoruba population is 42%, Kenya Kikuyu 34%, and Tswana Bostwana 22% [77]. Additionally, the allelic frequency of CYP2B6*9 has been reported to be 55% in Congolese, 20% in South African Xhosa, and 37% in Cameroonian populations [77].

Allelic variants of CYP2B6 *5 and *7 are associated with decreased CYP2B6 expression and the risk of VPA-induced ADRs in heterozygous and homozygous individuals compared to CYP2B6*1 (wild type) [78]. On the contrary, the highly functional allelic variant CYP2B6*4 increases the enzymatic activity of this isoenzyme [78], which is associated with a high rate of the P-oxidation of VPA and the risk of therapeutic resistance.

### 5.3. The CYP2C9 Gene

**Figure 6:**
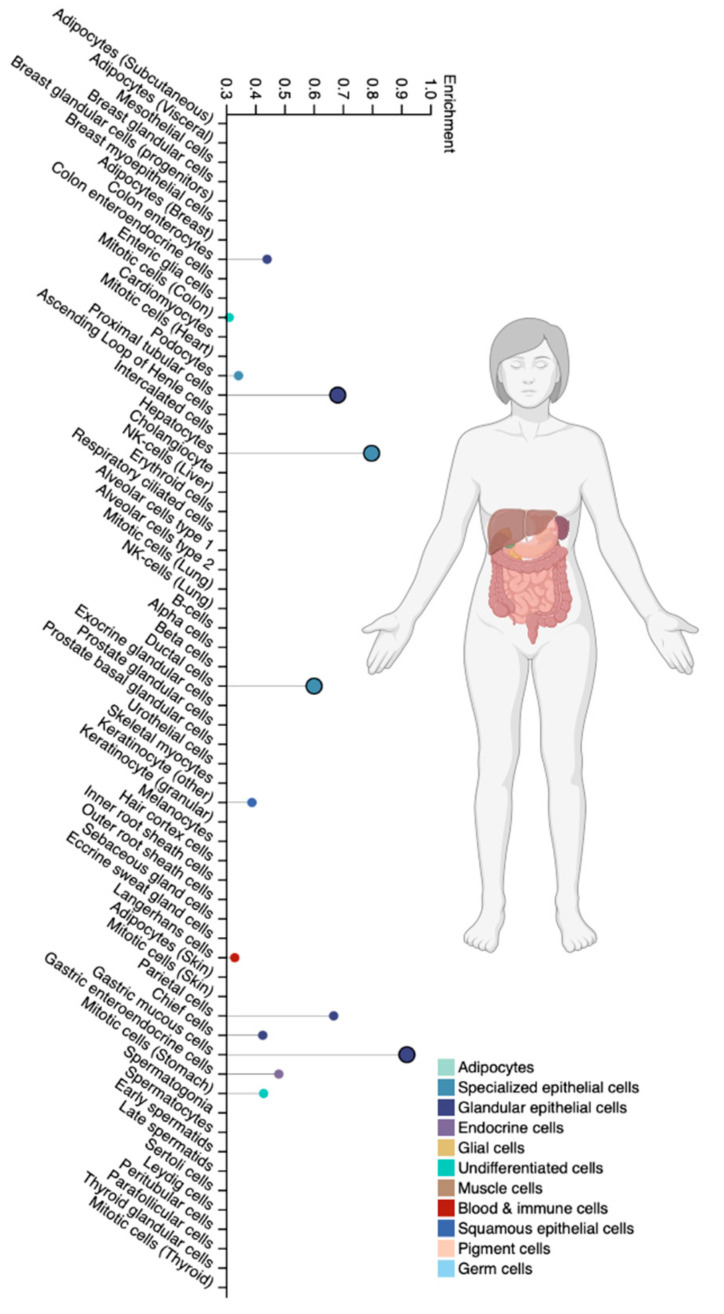
Tissue-specific cell types for the CYP2C9 isoenzyme (modified by the authors of The Human Protein Atlas [69]).

The CYP2C9 isoenzyme is cytochrome P450 family 2 subfamily C member 9, which is the most studied isoenzyme in the human CYP2C subfamily. It is expressed predominantly in the liver [67,80] (Figure 6) and is responsible for the metabolic clearance of up to 15–20% of all drugs undergoing phase I metabolism via P-oxidation, including VPA [67,80,81,82]. The participation of the CYP2C9 isoenzyme in the metabolism of VPA leads to the formation of its active metabolites VPA (for example, 2-ene-VPA, 4-ene-VPA, 4-OH-VPA, and 5-OH-VPA) [1,83]. At the same time, 2-ene-VPA has anticonvulsant effectiveness, and 4-ene-VPA, 4-OH-VPA and 5-OH-VPA have a high hepatotoxic potential [83,84,85].

The *CYP2C9* gene has nine exons and is transformed into 490 amino acids; it is located in the *CYP* gene cluster on chromosome 10 at position 10q24.33 (OMIM 601130) [80]. This gene is highly polymorphic, including fully functional allelic variants of great pharmaceutical and cogenetic significance. Changes in VPA P-oxidation caused by low-functional and non-functional allelic variants of the *CYP2C9* gene play an important role in the pathogenesis and severity of ADRs [81]. Individuals with one or two low-functioning alleles of the *CYP2C9* gene (IM and PM phenotypes, respectively) receiving conventional doses of VPA are at increased risk of developing serious ADRs (hepatotoxicity, neurotoxicity, metabolic syndrome) [80].

The most studied allelic variants of the *CYP2C9* gene are CYP2C9*2 (NC_000010.11:g.94942290C>T, p.R144C, rs1799853) and *3 (NC_000010.11:g.94981296A>C, p.I359L, rs1057910). In vitro, CYP2C9*2 reduces the activity of the isoenzyme by 50–70%, while CYP2C9*3 almost completely abolishes its function (75–99% reduction) [81,82]. In addition to the *2 and *3 allelic variants, several other variants may significantly affect CYP2C9 activity, including the low-functioning allelic variants CYP2C9 *5 (NC_000010.11:g.94981301C>G, p.D360E, rs28371686), *8 (NC_000010.11:g.94942309G>A, p.R150H, rs7900194), *11 (NC_000010.11:g.94981224C>T, p.R335W, rs28371685), and *14 (NC_000010.11:g.94942234G>A, p.R125H, rs72558189), and “null” alleles associated with a loss of function of the CYP2C9 isoenzyme (LOF) *6 (NC_000010.11:g.949283del, p.Lys273fs) [82].

An analysis of the frequencies of functionally significant alleles of the CYP family genes in 70 countries showed that the low-functional allele CYP2C9*2 is most common in European and Middle Eastern populations. However, allele frequency was the highest in Iran (incomplete allele frequency; MAF = 18.1%), Croatia (MAF = 16.5%), Lebanon (MAF = 15.4%), and France (MAF = 15%). In contrast, CYP2C9*2 was absent in East Asian populations and had a low allele frequency in South Asia (approximately 5%). In Africa, CYP2C9*2 was generally absent in sub-Saharan Africa, but allelic frequency was high in North African populations (up to 12%) [82]. In the Americas, high frequencies of CYP2C9*2 were observed in the Brazilian population (10.7%), but not in Ecuador (0.5%), Mexico (3.7%), and Peru (3.8%) [82].

Depending on ethnic groups, the allelic frequency of CYP2C9*2 is high in Sephardic Jews (MAF = 19.4%), the Jewish diaspora originating from the Iberian Peninsula, and Ashkenazi Jews (MAF = 13.5%), who are of Middle Eastern origin with evidence of European admixture [82]. Also, the allelic frequency of CYP2C9*2 is high among residents of Kosovo (MAF = 17.5%), while the frequency in the population of neighboring Serbia (MAF = 12.3%) and North Macedonia (12.4%) is lower [82]. The low-functioning allele CYP2C9*2 is mostly absent or rare in South and East Asian populations. Specific subpopulations, such as the Uyghurs (MAF = 7.8%) from Northwestern China and the Kannadiga ethnic group (MAF = 6%) from Southwestern India, have significantly higher allelic frequencies [82]. For the low-functioning allele CYP2C9*3, the highest frequencies (MAF = 36.2%) were found in the Jahai people, an indigenous population living in Malaysia [82]. Low-functioning SNVs CYP2C9*5, *6, *8 (c.449G>A, p.R150H, rs7900194), and *11 (c.1003C>T, p.R335W, rs28371685) are mainly found in people of African descent. It has been shown that the avoidance of PGx may lead to a significant overestimation of VPA dosage requirements in African Americans [80] due to the high allelic frequency of low-functioning and non-functional *CYP2C9* gene SNVs.

Global allelic frequencies of the non-functional allele of CYP2C9*3 coincide with the patterns of low-functional alleles. European and Middle Eastern populations have a high allelic frequency of CYP2C9*3, especially in Spain (10.1%) and Turkey (9.8%). However, this allelic variant was absent or rare in sub-Saharan African and East Asian populations. In addition, a very high frequency of CYP2C9*3 has been shown in the United Arab Emirates population (21.3%), which is in sharp contrast to other Middle Eastern populations where the frequency of CYP2C9*3 is about 6% [82]. CYP2C9*3 was very common in South Asian populations (with frequencies up to 11.9% in Pakistan and 11.6% in Bangladesh). In South American populations, the frequencies of CYP2C9*3 are relatively higher in Uruguay (7.6%), Colombia (6.8%), and Brazil (6%) but below 5% in all other countries in this region of the world [82]. Alleles *8 and *11 were common in populations of Africa and South America (mainly in Mozambique (14.6%) and Guarana (4.4%)). The *13 allele has been identified in East Asian and African American populations with a frequency ranging from 0.4 to 1.5%. At the same time, *5 and *6 alleles were most common in the United Arab Emirates (7.8%) and Sudan (2%) [82].

### 5.4. The CYP2C19 Gene

**Figure 7:**
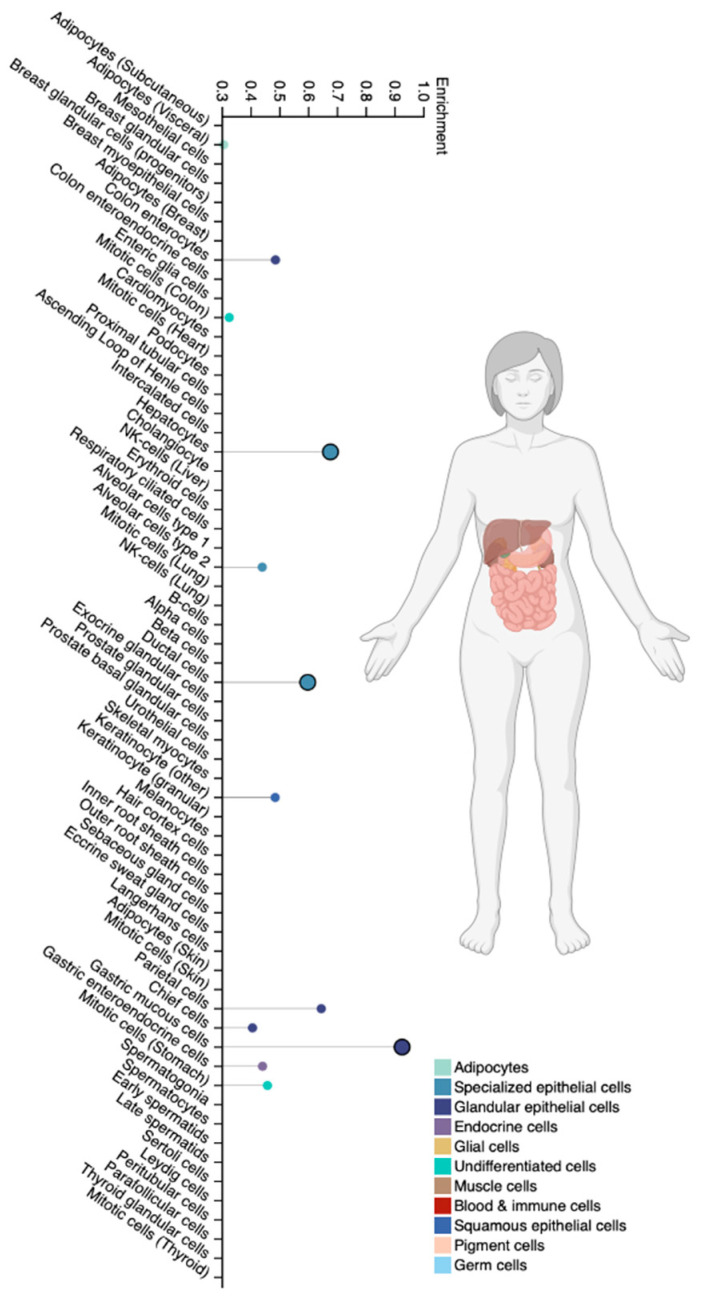
Tissue-specific cell types for the CYP2C19 isoenzyme (modified by the authors of The Human Protein Atlas [69]).

CYP2C19 is a cytochrome P450 family 2 subfamily C member 19. It is a clinically significant CYP isoenzyme and accounts for approximately 16% of the total CYP isoenzyme content in the liver (Figure 7). This protein is localized in the endoplasmic reticulum and is the main isoenzyme involved in the P-oxidation of VPA and other antiepileptic drugs (S-mephenytoin, diazepam, phenobarbital), as well as antimalarials (proguanil), oral anticoagulants (R-warfarin), chemotherapy drugs (cyclophosphamide), antiplatelet drugs (clopidogrel), proton pump inhibitors (omeprazole, pantoprazole, lansoprazole, rabeprazole), antiviral drugs (nelfinavir), and antidepressants (amitriptyline, clomipramine) [86,87].

The *CYP2C19* gene is localized on chromosome 10 at position 10q23.33 (OMIM 601130). About 35 SNVs in the *CYP2C19* gene have been studied [86]. Low-functioning allelic variants of CYP2C19*2 (rs4244285) and CYP2C19*3 (rs4986893) have been well studied. They are the most common and are associated with the pharmacogenetic profile of PM [88]. CYP2C19*2 (rs4244285) leads to a decrease in the activity of the isoenzyme. This SNV has a high allelic frequency among South Indians and Pacific Islanders and a low allelic frequency among European and African populations [88]. On the other hand, CYP2C19*3 (rs4986893) has a high allelic frequency in Japanese, Korean, and Chinese populations and a very low frequency in European and South African populations. Approximately 15–25% of the Southeast Asian population has a homozygous CYP2C19*3/CYP2C19*3 genotype and PM pharmacogenetic profile [88]. In contrast, the allelic variant CYP2C19*17 in the European population is associated with increased activity of the CYP2C19 isoenzyme, which can lead to rapid and ultra-rapid metabolism of VPA [88,89] in the liver, which is associated with a high risk of therapeutic resistance to VPA. The frequency of the CYP2C19*3 allele in the Iranian population is 1.7% [90]. The frequency of the CYP2C19*17 allele is 18–28% in European populations, 17–18% of Africans, and 0.3–4% of Asian populations, and among Iranian populations, it is 33.1–36.8% [90]. The frequency of CYP2C19*2 alleles among the African population is 16%, among the Caucasian population it is 13.3%, and among the Asian population it is 28.4%, respectively [86,90,91]. The frequency of CYP2C19*17 homozygotes is highest in Caspian populations and low in the Kurdish ethnic group [90].

The mean concentration/dose ratio of VPA was significantly higher in patients with one (*p* = 0.029) or two (*p* = 0.007) low-function alleles for the *CYP2C19* gene than in homozygous carriers of full-function alleles in an Asian population [24]. The mean VPA concentration/dose ratio was significantly higher in patients with the heterozygous *1/*2 genotype or the compound heterozygous *2/*3 genotype than in patients with the homozygous *1/*1 genotype. The genotypes studied play an important role in the control of steady-state VPA concentrations in blood serum [24] through TDM and studies of toxic VPA metabolites in biological fluids (blood, urine, saliva, etc.) [2].

### 5.5. The CYP2D6 Gene

**Figure 8:**
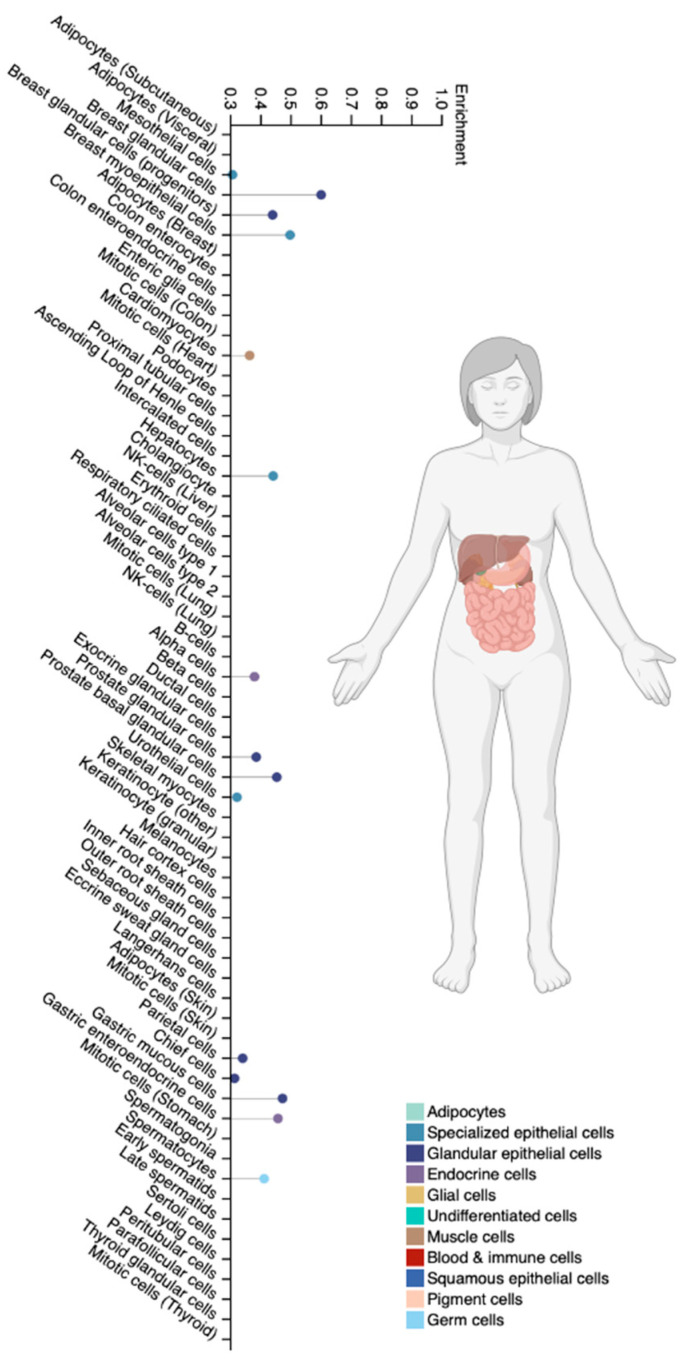
Tissue-specific cell types for the CYP2D6 isoenzyme (modified by the authors of The Human Protein Atlas [69]).

The CYP2D6 isoenzyme is a cytochrome P450 family 2 subfamily D member 6. CYP2D6 is expressed in the human gastrointestinal tract (Figure 8), but its abundance and catalytic activity in the small intestine is about one-fifth of that in the liver. The major contribution of gut-expressed CYP2D6 to drug metabolism is minor unless the substrate has a long residence time in the intestinal mucosa [92,93]. Despite the fact that the CYP2D6 isoenzyme makes up less than 5% of the CYP content in the liver, it is responsible for the metabolism of up to 25% of common drugs. In general, CYP2D6 is involved in the metabolism of drugs, such as VPA and other antiepileptic drugs, antipsychotics, antidepressants, beta-blockers, antifungals, antiretrovirals, antiarrhythmics, morphine, and tamoxifen derivatives. Many of these drugs have a narrow therapeutic window [94,95], which is important to remember to ensure their safety, especially when co-administered with VPA.

The *CYP2D6* gene is one of the most polymorphic genes of the CYP superfamily in humans, located on chromosome 22 at position 22q13 (OMIM 124030) [67]. About 80 different allelic variants have been identified and 130 genetic variations have been described [94]. These *CYP2D6* gene variants affect mRNA transcripts and alter proteins and enzyme catalytic activity. This is associated with a change in the rate of the P-oxidation of VPA in the liver.

Overall, CYP2D6 is a particularly challenging isoenzyme to understand and study due to its genetic polymorphism. Allelic variants in the *CYP2D6* gene demonstrate that some patients have no enzyme, some have low enzyme activity with only one active allele, some have two active alleles, and some have duplicate genes. Clinically, this genetic heterogeneity leads to the formation of EM, PM, and URM phenotypes [96]. The participation of the CYP2D6 isoenzyme in the metabolism of VPA has been shown in 1–8% of patients [97]. Full-function alleles CYP2D6*1 and CYP2D6*2 are associated with normal isoenzyme activity (EM phenotype). The two most important null (non-functional) allelic variants, CYP2D6*4 (rs3892097) and CYP2D6*5 (rs5030655), are associated with an inactive isoenzyme or absence of the isoenzyme, respectively. CYP2D6*4 and CYP2D6*5 (allelic frequencies of approximately 20 to 25% and 4 to 6%, respectively) are predominantly found in Caucasians [98]. A significant decrease in isoenzyme activity is also associated with allelic variants of CYP2D6*10 (rs1065852), CYP2D6*17 (rs28371706, c.2850C>T, rs16947), and CYP2D6*41 (rs28371725), and carriers of these SNVs have the PM phenotype. The predominant SNVs in people of Asian and African ancestry are CYP2D6*10 (allelic frequency about 50%) and CYP2D6*17 (allelic frequency about 20 to 34%), respectively. Both of these SNVs are associated with the formation of the PM phenotype [98]. Although the classical allelic frequencies determined in Asians (0 to 1% of the population) and Africans (0 to 5% of the population) are lower than in Caucasians (5 to 14% of the population), the high allelic frequency of CYP2D6*10 and CYP2D6*17 in these two populations explains the high prevalence of patients with the PM phenotype and provides a biological and molecular explanation for the use of lower dosages of VPA in people of Asian and African descent [98]. Statistically significant interregional differences in the frequency of carriage of risk allelic variants of the *CYP2D6* gene in Russia were shown. Patients with the IM phenotype (genotype *1/*10) predominated in the population of Samara, and patients with the RM phenotype (genotype *4/*10) predominated in the population of Krasnoyarsk. At the same time, the CYP2D6*1 allele, associated with the normal activity of this isoenzyme, was dominant in all groups of patients. According to TDM data, compound heterozygotes had higher VPA concentrations compared to the *1/*1 and *1/*10 genotypes (patients with the *1/*4 genotype and homozygotes for risk variants were absent in the studied samples). However, no significant association of the studied alleles and genotypes with the effectiveness of VPA therapy was found in the studied populations of Caucasians in Russia [97].

Also, the PM phenotype is associated with low-functioning allelic variants CYP2D6*9, *29, and *36 [98].

Significant interethnic heterogeneity in the distribution of studied *CYP2D6* gene alleles and variable phenotypes in patients with neurological diseases and mental disorders are well documented [96,99,100,101,102], which is important to consider for the development of PGx panels for different populations around the world.

## 6. Discussion

Despite the fact that the safety of valproate use, which is associated with the individual rate of its metabolism in the liver (in particular, P-oxidation) in a particular patient, is the result of the coordinated work of several cytochrome P450 isoenzymes and hence the normal functioning of several genes, the carriage of non-functional allelic variants (also known as risk alleles) is the cause of the development of serious VPA-induced ADRs. Often, the presence of these alleles leads to serious errors in the metabolism of drugs and is rejected in the process of evolution—patients with such risk alleles do not survive and/or have fertility disorders [3]. However, some clinically significant non-functional allelic variants of *CYP* superfamily genes persist in populations of various regions (countries) of the world and accumulate in the genome as heterozygotes. At the same time, some non-functional and low-functional allelic variants of CYPs may not manifest clinically immediately after the start of VPA administration. For example, a genetic predisposition to the impaired P-oxidation of valproates in the liver and the development of VPA-induced ADRs is most often detected when using this drug for a long time (3 months or more) and/or in high doses [3,20,31]. This explains why such allelic variants of *CYP* family genes may not be rejected in any way in vivo, and, therefore, their allelic frequency may be high in some populations. Naturally, homozygous carriers of minor (rare or, according to the new nomenclature, variable) non-functional alleles (risk alleles) of *CYP* genes are found quite rarely in the population, and heterozygous carriers of some clinically significant risk alleles may remain undiagnosed for a long time.

The results of genetic association studies convincingly demonstrate the role of the above-mentioned SNPs of the *CYP* family genes and the role of PGx in the promotion and safety of VPA in patients of other ethnic groups. There are no large-scale (multicenter) international studies devoted to the cost-effectiveness of introducing PGx into real clinical practice in neurology and psychiatry. Although dosing recommendations for many psychotropic medications, including VPA, are being developed based on PGx results, the valproates are prescribed to patients with psychiatric disorders who have not undergone predictive PGx and/or genetic screening [103,104]. TDM of plasma levels of VPA has been used in neurology and psychiatry for a long time [105]. This diagnostic method takes into account all factors of interindividual variability in the metabolism of VPA and other psychotropic drugs. However, PGx can be performed before VPA is prescribed, whereas TDM can only be performed when VPA and its reactive metabolites levels reach a stable level and when the patient may already have VPA-induced ADRs. Therefore, ideally, the neurologist or psychiatrist should consider all available genetic, physical, dietary, and environmental parameters to make the best possible choice of VPA and dosage when initiating therapy for each individual patient. Once the level of ingested VPA reaches a stable blood level, the study of TDM and active VPA metabolites [2] may be useful to clarify whether ADRs are due to disruption of the pharmacokinetics of VPA and its accumulation [1,37,106].

Ideally, PGx-based decision-making algorithms for the mental health practitioner should include information on a wide range of ethnic genetic, pharmacokinetic, and environmental factors associated with the risk of developing VPA-induced ADRs. This is a pressing interdisciplinary problem since many of the most reliable predictors of the risk of VPA-induced ADRs may be not only clinical but also psychosocial in nature [2,107].

However, most neurologists and psychiatrists express the opinion that it is advisable to prescribe PGx to patients with mental disorders in cases of the development of VPA-induced ADRs, followed by pharmacogenetic counseling [108,109]. At the same time, the developers of PGx panels support the predictive use of this diagnostic method (that is, before prescribing VPA) [110,111]. The reality is that the evidence base for both proactive and reactive use of PGx to predict and diagnose VPA-induced ADRs remains limited. Various decision systems are available to clinicians when choosing PGx. One of the most well-known systems is the Oxford Center for Evidence-Based Medicine (CEBM) level of evidence [112], which can help clinicians identify and assess the risks of VPA use [113].

Pharmacogenetically informed pharmacometabolomics is a new approach that allows individual prediction of the risk of impaired VPA P-oxidation, increased levels of active toxic VPA metabolites in biological fluids (blood, urine, saliva, etc.) based on unchanged PGx results, and an optimized selection and volume of metabolic studies (TDM and gas–liquid chromatography/mass spectrometry) [114]. In addition, it is promising and important in the future to compare the results of pharmacogenomic and pharmacometabolic studies of the therapeutic and toxic metabolites of VPA in order to identify allelic variants that contribute to metabolomic variations in different populations involving the frequencies of low-functioning and non-functional alleles of CYP family genes that are not influenced by the therapeutic response to valproate [1] in various ethnic and racial groups in the world population.

**Figure 9:**
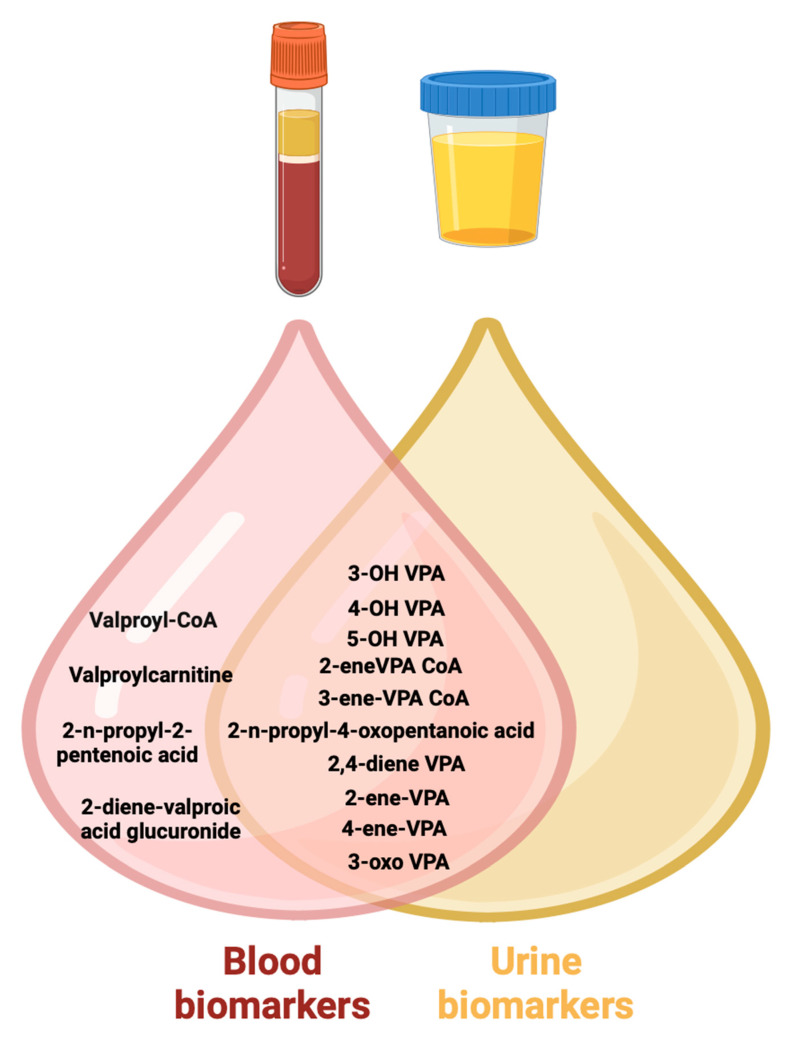
Toxic metabolic biomarkers of valproic acid in urine and blood (created with BioRender.com: https://www.biorender.com/ (accessed on 12 October 2023)). Note: VPA—valproic acid; 3-OH VPA—3-hydroxyvalproic acid; 4-OH VPA—4-hydroxyvalproic acid; 5-OH VPA—5-hydroxyvalproic acid; 2,4-diene VPA—2,4-diene valproic acid; 2-ene-VPA—2-ene-valproic acid; 4-ene-VPA—4-ene-valproic acid; 3-oxo VPA—3-oxovalproic acid; 2-ene-VPA CoA—2-ene-valproic acid coenzyme A; 3-ene-VPA CoA—3-ene-valproic acid coenzyme A; valproyl-CoA—valproyl-coenzyme A.

In conclusion, the carriage of non-functional and low-functioning alleles in different ethnic and racial groups may determine variable risks of the accumulation of toxic VPA metabolites (Figure 9) in the body of patients receiving long-term VPA and the risk of developing serious ADRs.

In 2011, a personalized approach to pharmacotherapy, called pharmacometabolic-informed pharmacogenomics, was proposed, which aims to identify metabolic biomarkers as a result of treatment [115]. It represents one of the promising strategies at the intersection of pharmacometabolomics and pharmacogenomics and may provide the key to understanding the mechanisms of the formation, action, and disposal of toxic VPA metabolites. However, significant limitations must be recognized in the application of this personalized approach to predicting safety and assessing the risk of adverse effects of VPA and its metabolites. First, VPA and most of its reactive metabolites are unstable compounds, requiring special technical conditions for sample collection and storage [116]. Ethnicity-formed pharmacometabolomics makes it possible to predict the interindividual safety and effectiveness of VPA therapy before starting valproate or within the first weeks of starting therapy. The prescription of VPA is not recommended for patients with the PM phenotype, although it is possible to prescribe it in a short course at a dose 50% lower than the average therapeutic one. The prescription of VPA is possible for patients with the IM phenotype, but the dosage of valproate should be reduced by an average of 25% of the average therapeutic one. At the same time, it is not recommended to additionally prescribe other drugs with a similar pathway of P-oxidation in the liver and/or drugs that are inhibitors of low-functioning CYPs in a particular patient [114].

It is possible to prescribe VPA in an average therapeutic dosage for a long time to patients with the EM phenotype. Finally, a dose increase of 25–50% of the average therapeutic dose is recommended for patients with URM phenotypes, depending on heterozygous or homozygous carriage of highly functional SNV gene(s) encoding CYPs involved in the metabolism of this drug.

Patients with the PM and IM phenotype, as well as patients with the EM profile (in the case of taking high doses of VPA, chronic use of valproate or polytherapy), require dynamic TDM at least once every 3 months, and a study of toxic pharmacometabolic biomarkers is presented in Figure 9. In addition, toxic metabolites can be identified in other biological fluids in the human body, including saliva [117,118], sweat, [119] exhaled air [120], cerebrospinal fluid [121], and interstitial fluid [122]. However, pharmacometabolic studies of VPA are still in the development stage and are not used in real clinical practice in most countries [123].

## 7. Conclusions

This narrative review demonstrated the importance of considering the allelic frequencies of the *CYP* family genes encoding key isoenzymes of the P-oxidation of VPA and its compounds in different populations around the world. PGx panels are highly informative and specific in one ethnic/racial group but may be uninformative or poorly informative in another ethnic/racial group. This problem is very relevant for countries with large ethnic heterogeneity of populations. Large randomized clinical trials are necessary to clarify the relationship between the carrier of variable alleles of genes encoding key isoenzymes of the P-oxidation of VPA and serum levels of VPA in various ethnic/racial groups since other factors may affect the metabolic rate of this drug in the liver (dietary characteristics, comorbid liver diseases, age, drug–drug interaction). Although P-oxidation accounts for a minor part of the VPA metabolic pathway, it is important for toxicity in patients with impaired UGTs. Because of the inconsistent results about the influences of CYP genetic variants on VPA pharmacokinetics, depending on the ethnicity of the patients, larger cohorts may be needed to verify these results and examine newer candidate genes.

## Figures and Tables

**Table 1:** Modifiable and non-modifiable risk factors for the impaired P-oxidation of valproic acid and the development of adverse drug reactions.

Modifiable Risk Factors	Non-Modifiable Risk Factors
High doses of valproic acid	Heredity (monogenic hereditary liver and metabolic diseases)
Long-term use of valproic acid	Genetic predisposition to the impaired P-oxidation of valproic acid (IM and PM phenotypes)
Polypharmacy (simultaneous prescription of five or more drugs, especially those with a similar metabolic pathway)	Gender (female patients)
Poor nutrition	Age (elderly patients)
Stress	Ethnic and racial background of patients
Sleep disturbance	
Smoking	Comorbid illnesses and mental disorders
Alcohol abuse Low physical activity (sedentary lifestyle)	Duration of the disease Liver failure
Polytherapy (simultaneous administration of VPA and other psychotropic drugs with a similar metabolic pathway)	

**Table 2:** Frequency of non-functional and low-functional single nucleotide variants associated with P-oxidation disorders in populations of different regions of the world.

Isoenzyme and Tissue Expression Claster (Location) **	Gene (OMIM), Location ***	Single Nucleotide Variant (RS ID) ****	Allele Frequency *				
			North America	South America	Asia	Africa	Europe
CYP2A6 (cytochrome P450 family 2 subfamily A member 6). Liver—plasma proteins (mainly). Intracellular	*CYP2A6* (122720), 19q13.2	rs1801272	0.02	0.01	0.01	-	0.03
		rs111033610	-	-	0.01	-	-
		rs28399447	-	-	0.01	-	-
		rs4986891	0.01	-	-	0.045	-
		rs28399433	0.09	0.09	0.19	0.08	0.06
		rs28399447	-	-	0.01	-	-
		rs28399454	0.007	0.01	0.004	0.12	-
		rs1809810	0.01	0.01	0.02	0.01	0.02
		rs72549435	0.007	0.005	0.004	0.01	-
		rs59552350	0.015	-	-	0.004	-
		rs4986891	0.015	-	-	0.004	-
		rs28399445	-	0.01	-	0.01	-
CYP2C9 (cytochrome P450 family 2 subfamily C member 9). Liver—plasma proteins (mainly). Intracellular	*CYP2C9 *(601130), 10q23.33	rs1799853	0.09	0.12	0.02	-	0.14
		rs1057910	0.03	0.03	0.1	-	0.08
		rs28371686	0.02	0.01	-	0.01	-
		rs7900194	0.02	0.04	0.005	0.05	0.004
		rs28371685	0.007	0.01	0.004	0.02	0.01
		rs72558187	-	-	0.04	-	-
CYP2B6 (cytochrome P450 family 2 subfamily B member 6). Liver—plasma proteins (mainly). Intracellular	*CYP2B6* (123930), 19q13.2	rs12721655	0.01	0.005	0.005	-	-
		rs28399499	0.005	0.02	-	0.07	-
		rs36079186	-	-	0.005	0.01	-
CYP3A4 (cytochrome P450 family 3 subfamily A member 4). Liver—metabolism (mainly). Membrane; intracellular	*CYP3A4* (124010), 7q22.1	rs4986910	0.01	0.01	-	-	0.01
		rs28371759	-	-	0.02	-	-
CYP2C19 (cytochrome P450 family 2 subfamily C member 19). Liver—metabolism (mainly). Intracellular	*CYP2C19* (124020), 10q23.33	rs4244285	0.12	0.11	0.3	0.18	0.1
		rs28399504	0.007	0.0004	0.0004	-	0.0004
		rs41291556	0.01	-	-	-	-
CYP2D6 (cytochrome P450 family 2 subfamily D member 6). Liver—plasma proteins (mainly). Membrane	*CYP2D6*, (124030) 22q13.2	rs3892097	0.11	0.12	0.12	0.05	0.16
		rs5030865	-	-	0.01	-	-
		rs1065852	0.14	0.16	0.31	0.1	0.19
		rs28371706	0.01	0.01	-	0.2	-

Note: [65] *; [66] **; [67] ***; [68] ****. Median value determined by maf (minor allele frequency).
